# Phenotypical variability of the sigmoid sinus in translabyrinthine and retrosigmoid surgeries

**DOI:** 10.1007/s00276-022-02988-7

**Published:** 2022-07-24

**Authors:** Maryna Al-Fauri, Daniel Lee, Paul Kelly

**Affiliations:** Basic Science Department, AUC School of Medicine, Philipsburg, Sint Maarten

**Keywords:** Sigmoid sinus, Retrosigmoid approach, Translabyrinthine approach, Cranial phenotypes, Variability

## Abstract

**Introduction:**

We hypothesized that the cranial phenotype influences the shape of the posterior cranial fossa and the relative position of the sigmoid sinus.

**Materials and methods:**

The topography of the sigmoid sinus was studied on 26 magnetic resonance venograms and 35 embalmed cadavers by morphometric analysis, dissection, and photo modeling techniques.

**Results:**

The data show that the transverse diameter of the posterior cranial fossa correlates positively with the laterolateral diameter of the skull. The majority of cases with the low-anterior position of the sigmoid sinus were recorded in the brachycephalic group (82%), while the high-posterior localization of the sigmoid sinus was typical for the dolichocephalic patients (63%). The results of the ANOVA test confirm the significance of differences.

**Conclusions:**

The shape of the skull reflects the morphology of the posterior cranial fossa and influences the topographic characteristics of the sigmoid sinus that must be considered in the selection of surgical approach to the inner ear and pontocerebellar angle.

## Introduction

The position of the sigmoid sinus is an important limiting factor in translabyrinthine and retrosigmoid approaches to the pontocerebellar angle and structures of the inner ear. High-posterior localization of the sinus influences the diameter of the surgical wound and increases the degree of cerebellar retraction in retrosigmoid surgeries [5, 8, 10]. The low-anterior position of the sinus complicates the translabyrinthine approach to the structures of the inner and middle ear by narrowing the operative window [2, 4, 13]. The diameter, shape, and position of the sigmoid sinus are carefully considered in choosing the surgical approach to the structures of the posterior cranial fossa and inner ear.

The positional relationships of the sigmoid sinus with the surrounding intra- and extracranial structures are reported by several authors. The statistical differences for the distances between the various parts of the sigmoid sinus and posterior semicircular canal, facial nerve, internal and external acoustic meatuses for males and females, and the right and left sides of the head are available in the literature [2, 12, 14]. Several bony landmarks and laborious coordinate systems aimed to assist in localizing the sinus along were proposed by authors [3, 6]. However, the influence of the shape of the skull on the position of the sigmoid sinus has never been reported.

We assume that the cranial phenotype does influence the shape of the posterior cranial fossa and determines the relative position of the sigmoid sinus. This research aims to study the topography of the sigmoid sinus and its positional relationships with the external and internal acoustic meatuses in subjects with brachycephalic, mesocephalic, and dolichocephalic skulls.

## Materials and methods

Twenty-six magnetic resonance venograms of the head (32–69 years, 10 males, 16 females) and 35 cadavers (54–78 years, 17 males, 18 females) without known osteological pathology and visible head deformation were included in this study. All body donors were legally competent and had a will in which they agreed to the use of their body for research, study, or teaching purposes; the patients who underwent radiologic examination have signed the written consent. The institutional review board has approved the study.

### Neuroimaging data

**Fig. 1:**
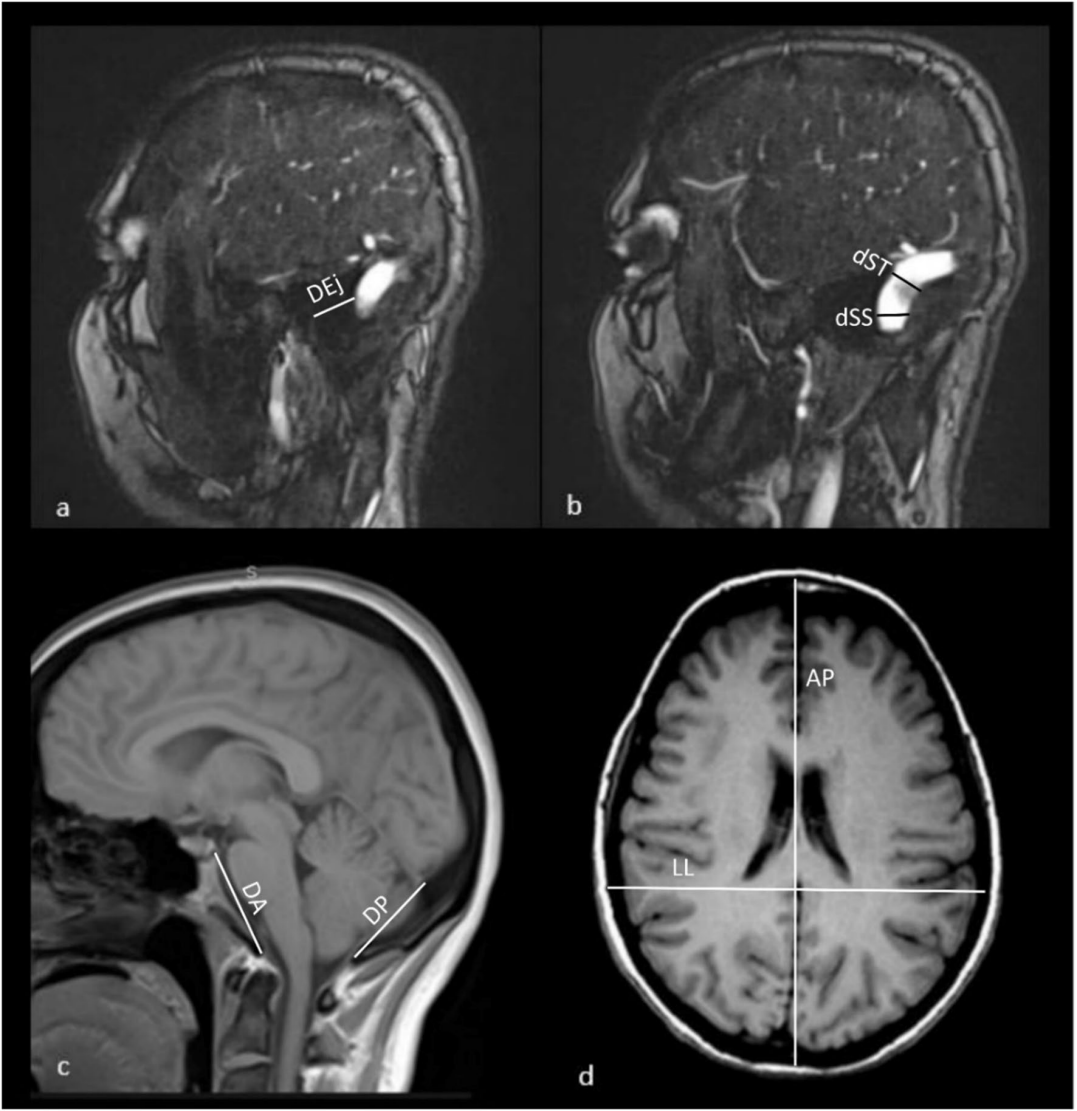
Magnetic resonance venograms of the head demonstrate the measurements taken: **a** shortest distance between the posterior margin of the external acoustic meatus and the transverse-sigmoid sinus junction (DEj); **b** diameter of the transverse-sigmoid sinus junction and the diameter of the sigmoid sinus (dST and dSS, respectively); **c** distance between the internal occipital protuberance and the posterior margin of the foramen magnum (DP); the distance between the dorsum sellae and the anterior margin of the foramen magnum (DA); **d** latero-lateral (LL) and the anteroposterior (AP) diameters of the skull. The RadiAnt Pro (2021) software was employed to analyze the imaging data

On the parasagittal magnetic resonance venograms (MRVs) we identified the external acoustic meatus (EA) and the transverse-sigmoid sinus junction (TSj) and measured the distance between them (Fig. 1a). The diameters of the transverse-sigmoid sinus junction (dTS) and the vertical part of the sigmoid sinus (dSS) were recorded as well (Fig. 1b). Subject to the relative position of the sigmoid sinus, all specimens were classified into three types as previously described by Satoshi Tsutsumi et al. [14]. To assess the morphology of the skull and posterior cranial fossa, the distances between the following landmarks were measured:

- Glabella and external occipital protuberance of the skull (AP),
- Right and left parietal eminences of the skull (LL),
- Right and left asterion (pLL),
- Dorsum sellae and external occipital protuberance (pAP),
- Dorsum sellae and the anterior margin of the foramen magnum (DA),
- Internal occipital protuberance and the posterior margin of the foramen magnum (DP) (Fig. 1c, d).

### Cadaveric data and 3D modeling

The cadavers underwent routine educational dissection in the specialized laboratory of our organization. After the dissection of the superficial tissues of the head and craniotomy, the brain and meninges were removed from the cranial cavity exposing the skull base. Then, the cadaveric specimens were digitized using the Photo Modeler (2021) software and technique, as previously described [1].

**Fig. 2:**
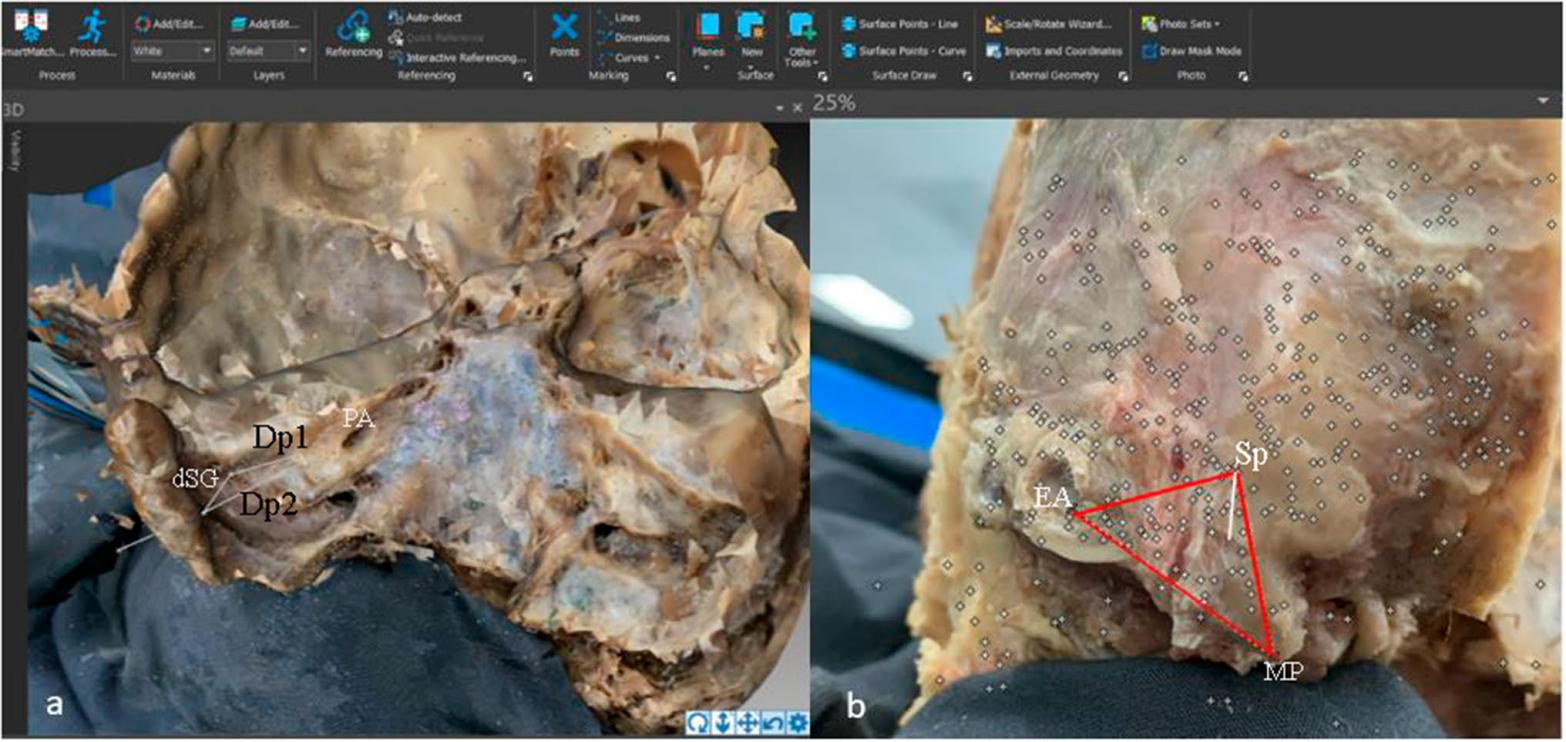
Working window of the photo modeler platform with a skull base model is shown in this picture (Photo Modeler, 2021). All the reference points were set up in a plane parallel to the superior border of the pyramid of the temporal bone. After measuring the diameter of the sigmoid groove (dSG), the distances from its anterior (Dp1) and posterior (Dp2) margins to the porus acusticus (PA) were taken. Then, the Dp2 line was extended to the outer surface of the temporal bone (red arrow), and the distances between the posterior wall of the external acoustic meatus (EA), the tip of the mastoid process (MP), and the projected posterior margin of the sigmoid groove (Sp) were measured accordingly

On the obtained 3D models, the following intra- and extracranial distances were taken digitally (Fig. 2)

- Between the lateral border of the porus acusticus and the anterior margin of the sigmoid groove (Dp1)
- From the lateral border of the porus acusticus to the posterior margin of the sigmoid groove (Dp2)
- Anteroposterior diameter of the sigmoid groove (dSG)
- From the external acoustic meatus to the tip of the mastoid process (Pp1)
- Between the external acoustic meatus and the projection point of the posterior margin of the sigmoid groove (Pp2)
- Between the tip of the mastoid process and the projection point of the posterior margin of the sigmoid groove (Pp3)
- From the glabella to the external occipital protuberance of the skull (AP)
- Between the right and left parietal eminences of the skull (LL)

### Cranial phenotype

The cranial index (CI) was calculated as a percentage ratio of the laterolateral diameter of the skull to the anteroposterior diameter [11]. All specimens were subdivided into 3 groups according to the CI value: dolichocephalic (CI < 74.9), mesocephalic (CI = 75–79.9), and brachycephalic (CI > 80).

### Statistical analysis

The methods of descriptive statistics were used to analyze the average and distribution of the variables in each phenotypical and gender group. The Pearson’s correlation was applied to evaluate the influence of the diameters of the skull on the variables. Analysis of variance (ANOVA) was employed to verify the significance of differences in the cranial phenotypical groups (SPSS software package, 2020).

## Results

### Analysis of the data obtained from the magnetic resonance venograms

Morphology of the posterior cranial fossa reflects the general shape of the skull. The data in Table 1 shows that the anteroposterior diameter of the posterior cranial fossa peaks in dolichocephalic specimens, while the laterolateral diameter prevails in the brachycephalic ones. The posterior cranial fossa was deeper in patients with dolichocephalic skulls compared to the other cranial phenotypes (Table 1). The transverse-sigmoid sinus junction was located further from the external acoustic meatus in patients with dolichocephalic skulls compared to the other cranial phenotypes (Table 1). The majority of cases with low and extremely approximated to the external acoustic canal position of the sinuses were recorded in the brachycephalic group (82%), while the higher localization of the transverse-sigmoid sinuses was typical for the dolichocephalic patients (63%) (Table 2). The outer diameter of the transverse-sigmoid sinus junction was comparatively smaller in the brachycephalic patients; the diameter of the vertical part of the sigmoid sinus showed the same tendency, although the significance of differences was low.

**Table 1:** Descriptive statistics and analysis of variants of the structures of the posterior cranial fossa taken from magnetic resonance venograms (mean, ± SD, mm)

Variables	Males (*n* = 20)	Females (*n* = 32)	DC (*n* = 10)	MC (*n* = 16)	BC (*n* = 26)
DEj	12.035 (3.024)	10.31 (2.41)	12.91 (3.68)	11.02 (2.38)	9.19 (2.41)
	0.034*		0.051*		
dST	11.94 (1.71)	10.69 (2.58)	11.59 (1.35)	10.58 (2.14)	10.06 (2.45)
	0.706*		0.049*		
dSS	9.66 (1.96)	10.56 (2.58)	10.23 (2.83)	10.17 (2.30)	10.02 (1.78)
	0.151*		0.377*		
pLL	114.04 (4.34)	105.6 (2.25)	107.86 (2.91)	110.4 (0.56)	112.6 (0.27)
	0.0001*		0.002*		
pAP	79.02 (4.73)	77.97 (5.12)	81.42 (2.59)	78.30 (4.47)	75.40 (4.16)
	0.465*		< 0.001*		
DP	39.13 (2.25)	39.60 (5.32)	41.88 (5.61)	40.06 (3.91)	37.70 (3.43)
	0.711*		0.024*		
DA	39.71 (4.02)	37.12 (4.29)	39.72 (2.48)	38.24 (2.71)	34.67 (5.89)
	0.038*		0.004*		

*DEj* the distance between the external acoustic meatus and the transverse-sigmoid sinus junction, *dST* the diameter of the transverse-sigmoid sinus junction, *dSS* the diameter of the sigmoid sinus, *pLL* the laterolateral diameter of the posterior cranial fossa**, ***pAP* the anteroposterior diameter of the posterior cranial fossa**, ***DP* the distance from the internal occipital protuberance to the posterior margin of the foramen magnum**, ***DA* the distance from the dorsum sellae to the anterior margin of the foramen magnum

^*^Significance of differences of the variants in the compared groups (ANOVA)

**Table 2:** Topographical characteristics of the transverse-sigmoid sinus junction in relation to the external acoustic meatus in brachycephalic (BC), dolichocephalic (DC), and mesocephalic (MC) phenotypical groups

Variables	DC (*n* = 10)	MC (*n* = 16)	BC (*n* = 26)
Type I (*n* = 11)	7 (63%)	2 (18%)	2 (18%)
Type II (*n* = 29)	3 (10.3%)	14 (48.27%)	12 (41.37%)
Type III (*n* = 11)	0	2 (18%)	9 (82%)

For visualization of the presented numerical data see Fig. 4

*DC* dolichocephalic, *MC* mesocephalic, *BC* brachycephalic, *Type I* localization of the transverse-sigmoid sinus junction above the external acoustic meatus (as seen on the parasagittal MR venograms), *Type II* localization of the transverse-sigmoid sinus junction at the same level with the external acoustic meatus, *Type III* the transverse-sigmoid sinus junction lies below than the acoustic meatus (classification by Satoshi Tsutsumi et.al.)

The correlation analysis presented in Table 3 confirms the tendencies highlighted above. The data show that the distance between the external acoustic meatus and the transverse-sigmoid sinus junction correlates positively with the anteroposterior diameter of the skull. In addition, the vertical diameters of the posterior cranial fossa correlate positively with the anteroposterior diameter of the posterior cranial fossa and negatively with the laterolateral one.

**Table 3:** Correlation between the variables of the posterior cranial fossa measured on the magnetic resonance venograms, *n* = 52, PC (two-tailed)

	(1)	(2)	(3)	(4)	(5)	(6)	(7)	(8)	(9)
(1) LL	1								
(2) AP	0.373^**^	1							
(3) DEj	0.086	0.279^*^	1						
(4) dSG	− 0.124	0.193	0.030	1					
(5) dSS	− 0.150	− 0.057	0.107	0.372^**^	1				
(6) pLL	0.568^**^	0.104	0.176	− 0.068	− 0.128	1			
(7) pAP	− 0.171	0.396^**^	0.004	0.325^*^	0.030	− 0.272	1		
(8) DP	− 0.221	0.071	0.332^*^	0.039	− 0.064	− 0.531*	0.305	1	
(9) DA	0.271	− 0.152	0.189	− 0.112	− 0.320^*^	− 0.230	0.437^**^	0.341^*^	1

*LL* the laterolateral diameter of the skull**, ***AP* the anteroposterior diameter of the skull**, ***DEj* the distance between the external acoustic meatus and the transverse-sigmoid sinus junction, *dST* the diameter of the transverse-sigmoid sinus junction, *dSS* the diameter of the sigmoid sinus, *pLL* the laterolateral diameter of the posterior cranial fossa**, ***pAP* the anteroposterior diameter of the posterior cranial fossa**, ***DP* the distance from the internal occipital protuberance to the posterior margin of the foramen magnum**, ***DA* the distance from the dorsum sellae to the anterior margin of the foramen magnum

**Correlation is significant at the 0.01 level (two-tailed)

*Correlation is significant at the 0.05 level (two-tailed)

On average, all variables were larger in males, but only the difference of laterolateral diameters of the posterior cranial fossa showed statistical significance (Table 1).

### Analysis of the data obtained from the digital models of the cadaveric skull bases

Overall, the distances from the lateral border of the porus acusticus to the anterior and posterior margins of the sigmoid groove peaked in the dolichocephalic specimens (Table 4). The diameter of the sigmoid groove was larger in the mesocephalic group but revealed its smallest value in the brachycephalic specimens. On average, the projection point of the posterior edge of the sigmoid groove on the mastoid process was located approximately 5 mm further from the external acoustic meatus in the dolichocephalic group compared to the brachycephalic one. The distance between the tip of the mastoid process and the external acoustic meatus was smaller in the brachycephalic skulls.

**Table 4:** Descriptive statistics and analysis of variants of the structures of the posterior cranial fossa obtained from the cadaveric specimens of the skull base (mean, ± SD, mm)

Variables	Males (*n* = 34)	Females (*n* = 36)	DC (*n* = 22)	MC (*n* = 18)	BC (*n* = 30)
Dp1	26.40 (3.64)	25.07 (3.43)	26.55 (4.91)	24.55 (2.85)	25.83 (2.55)
	0.125*		0.308*		
Dp2	37.55 (30.21)	35.34 (3.19)	38.40 (3.66)	37.09 (3.69)	36.33 (2.63)
	0.005*		0.104*		
dSG	12.42 (2.53)	12.14 (2.98)	12.11 (2.91)	13.60 (3.52)	11.58 (1.62)
	0.679*		0.047*		
Pp1	22.28 (3.97)	19.26 (3.01)	21.43 (3.64)	20.45 (2.67)	19.25 (4.24)
	0.001*		0.071*		
Pp2	34.10 (3.97)	32.14 (3.73)	34.72 (4.13)	31.65 (3.53)	29.15 (3.95)
	0.041*		0.045*		
Pp3	38.45 (6.10)	37.85 (6.64)	39.81 (6.69)	34.28 (5.57)	30.76 (5.92)
	0.704*		0.031*		

*Dp*1 the distance from the anterior margin of the sigmoid groove to the porus acusticus**, ***Dp*2 the distance from the posterior margin of the sigmoid groove to the porus acusticus**, ***dSG* the diameter of the sigmoid groove**, ***Pp*1 the distance between the external acoustic meatus and the tip of the mastoid process**, ***Pp*2 the distance between the external acoustic meatus and the projection point of the posterior margin of the sigmoid groove**, ***Pp*3 the distance between the tip of the mastoid process and the projection point of the posterior margin of the sigmoid groove

^*^Significance of differences of the variants in the compared groups (ANOVA)

The distances between the external acoustic meatus, the tip of the mastoid process, and the projection point of the sigmoid groove correlated positively with each other and negatively with the laterolateral diameter of the skull (Table 5). In addition, the data show that the posterior edge of the sigmoid groove has some mutual relationships with both internal and external acoustic meatuses, as those distances correlate with each other.

**Table 5:** Correlation between the variables of the posterior cranial fossa measured on the cadaveric specimens, *n* = 70, PC (two-tailed)

	(1)	(2)	(3)	(4)	(5)	(6)	(7)	(8)
(1) AP	1							
(2) LL	0.408^**^	1						
(3) Dp1	0.049	0.032	1					
(4) Dp2	0.261^*^	− 0.032	0.474^**^	1				
(5) dSS	0.189	− 0.030	− 0.325^**^	0.320^**^	1			
(6) Pp1	0.085	− 0.372^**^	0.137	0.221	0.050	1		
(7) Pp2	0.083	− 0.218^*^	0.188	0.412^**^	0.186	0.334^**^	1	
(8) Pp3	− 0.038	− 0.009	0.181	0.259^*^	0.125	0.292^*^	0.561^**^	1

*AP* the anteroposterior diameter of the skull, *LL* the laterolateral diameter of the skull, *Dp*1 the distance from the anterior margin of the sigmoid groove to the porus acusticus**, ***Dp*2 distance from the posterior margin of the sigmoid groove to the porus acusticus**, ***dSG* the diameter of the sigmoid groove**, ***Pp*1**,** the distance between the external acoustic meatus and the tip of the mastoid process**, ***Pp*2 the distance between the external acoustic meatus and the projection point of the posterior margin of the sigmoid groove**, ***Pp*3 the distance between the tip of the mastoid process and the projection point of the posterior margin of the sigmoid groove

**Correlation is significant at the 0.01 level (two-tailed)

*Correlation is significant at the 0.05 level (two-tailed)

All measurements were larger in males than in females (Table 1). The difference of distances between the external acoustic meatus and the tip of the mastoid process, the external acoustic meatus and the projection point of the posterior edge of the sigmoid groove, and between the porus acusticus and the posterior edge of the sigmoid groove were statistically significant (Table 4).

## Discussion

**Fig. 3:**
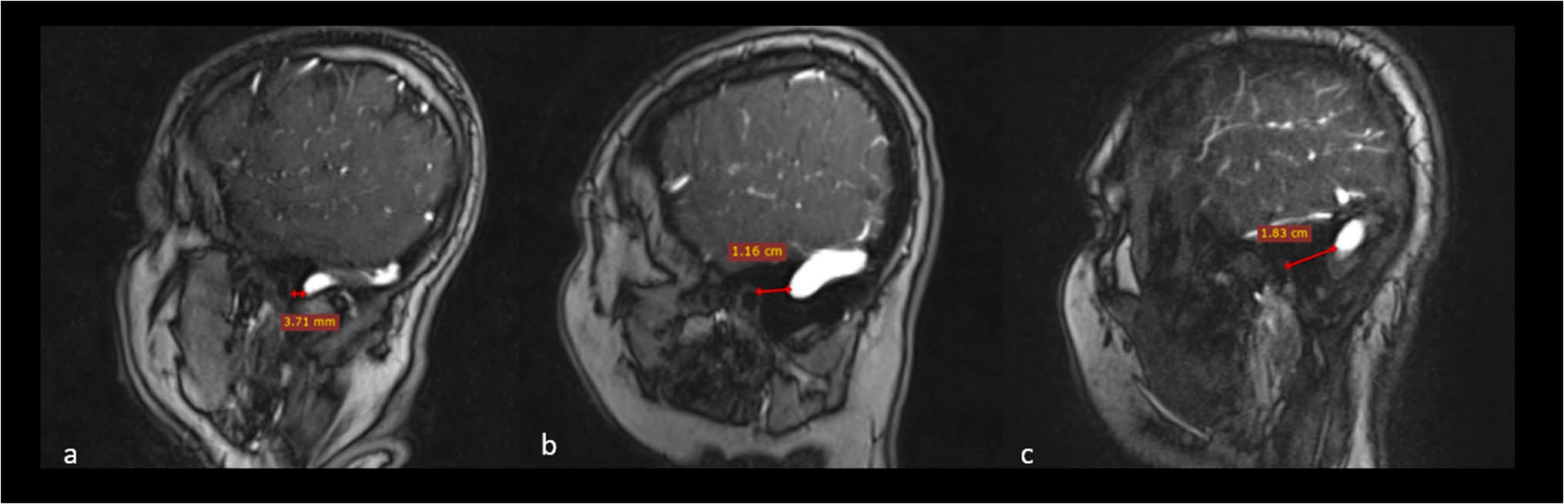
Sagittal magnetic resonance venograms of the head demonstrate the topographic relationship of the transverse-sigmoid sinus junction and the external acoustic meatus by shapes of the skull. The inferior position of the sinuses (Type III) picked in brachycephalic patients **(a)**, the superior position (Type I) dominated in the dolichocephalic patients **(c)**, while patients with the normal shape of the head **(b)** showed the prevalence of intervening localization of the transverse-sigmoid sinus junction. For precise numerical data see Table 5

**Fig. 4:**
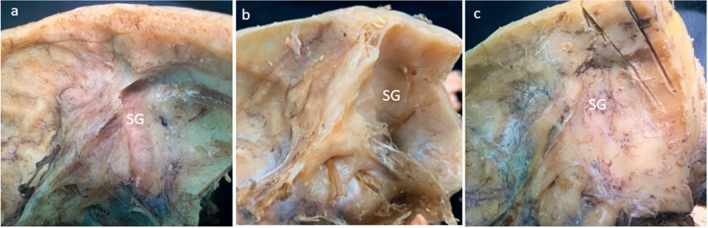
Cadaveric specimens of the skull base demonstrate the range of variability of the sigmoid groove (SG). Note the low localization and prominent indentation of the sigmoid groove into the pyramid of the temporal bone in specimen “**a**”, which belongs to a 68-year-old female with the cranial index (CI) equal to 83.6 percent. As an opposite, the shallow running up groove is barely visible on specimen “c” (a 56-year-old male, with CI = 72.1 percent). The specimen “**b**” belongs to a 63-year-old female with CI = 75.6 percent, and it shows the intermediate topography of the sigmoid groove

**Fig. 5:**
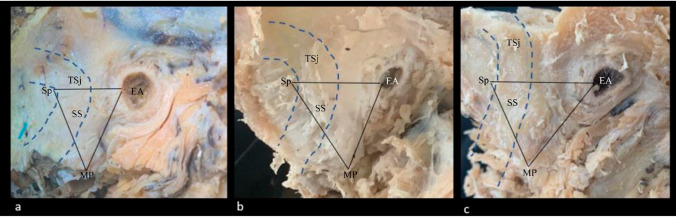
Summative scheme depicting the phenotypical characteristics of the topography of the sigmoid sinus (SS) and transverse-sigmoid sinus junction (TSj) projected on the mastoid process (MP) of the temporal bone. The transverse-sigmoid sinus junction is located lower and closer to the external acoustic meatus (EA) in the brachycephalic specimens **(a)** and is significantly higher and further from the EA in the dolichocephalic **(c)** ones. The mesocephalic heads **(b)** featured the intermediate position of the sinuses. The coordinates were collected and summarized using the photo modeling technique

We found that the transverse and longitudinal diameters of the posterior cranial fossa correspond to the phenotype of the skull. The depth of the posterior cranial fossa is shallower in brachycephalic patients and increases with the anteroposterior diameter of the skull. The obtained patterns of distribution of the distances between the external acoustic meatus and the transverse-sigmoid sinus junction by cranial phenotype allow us to suggest that the position of the sigmoid sinus is determined by the shape of the skull. The diameter of the sinus increases with the anteroposterior diameter of the posterior cranial fossa. In the dolichocephalic patients, the sigmoid sinus takes a more vertical course, while in the brachycephalic ones, it loops forward approaching the external acoustic meatus (Fig. 3). Such proximity of the sigmoid sinus often results in the indentation of the sigmoid groove into the base of the temporal pyramid limiting the possibilities for the translabyrinthine procedures on the cochlea and internal acoustic canal (Fig. 4). The posterior margin of the sigmoid groove is the most remote from both the porus acusticus and external acoustic canal in dolichocephalic specimens. That implements the wider retrosigmoid approach and increases the operative angle in the treatment of acoustic neuromas in this category of patients (Fig. 5). The findings on the shape of the posterior cranial fossa and the topography of the sigmoid sinus in different cranial phenotypes are novel.

Even though the individual variability of the skull base influences the flow and outcomes of surgeries on the basilar structures, the phenotypical characteristics of the skull base are scarcely addressed by authors, and the existing reports are controversial. For instance, Meneses et al. concluded that the distance between the outer surface of the skull and the apex of the temporal pyramid is shorter in brachycephalic skulls for both middle and posterior cranial fossa approaches due to narrowing of the anterior but widening of the posterior parts of the skull base in this phenotype [7]. The authors analyzed neither the diameters of the cranial fossae nor the orientation of the pyramid of the temporal bone but measured only the distances from the surface of the skull to the apex, however. The results of our early research showed that the length of the superior border of the pyramid of the temporal bone and the distance from the apex of the pyramid to the squama is larger in brachycephalic specimens due to the positive correlation of the laterolateral diameter of the middle cranial fossa with the transverse diameter of the skull [1]. The orientation of the temporal pyramid varied between the phenotypes significantly, with a half the size of the angle between the superior border of the pyramid and the squama in dolichocephalic specimens. Such distribution of variables indicates the approximation of the apex of the pyramid to the lateral wall of the middle cranial fossa in the specimens with narrow skulls contradicting the data presented by the authors [7].

The sigmoid sinus represents the major dural channel draining the entire cranial cavity. It runs along the inner surface of the temporal bone connecting the transverse sinus with the internal jugular vein. The intense venous flow carves the bone developing a tortuous groove in this location with age. Examining the inner surface of dry skulls, we see how variable the position of the sigmoid groove in relation to other structures of the temporal pyramid is (Fig. 4). To predict the localization of the sigmoid sinus in potential patients, authors advise using the external landmarks of the skull, such as asterion, temporoparietal suture, and mastoid process [2, 12]. Li et al. designed the entire coordinate system using the mastoid notch as an essential reference point [6]. Practical application of this method would require specific orientation of the patient’s head and extensive skull exposure during the procedure. None of the existing methods provided a solution to the problem of individual variability effectively. In the present research, we built a systematic approach that would allow surgeons to understand the tendency of the distribution of the variables and predict the individual features of the skull base structures based on the individual shape of the skull only.

Despite the wide implementation of radiologic methods, the evaluation of the relative position of the sigmoid sinus on CT and MR venograms is challenging as we can assess only one type of tissue at a time. To solve this problem, several radiologic landmarks and methods were proposed by the authors. Sheng et al. suggested merging the subtraction computed tomography angiography data with non-enhanced data and reducing the transparency of the bones to visualize the underlying venous channels [12]. Galal et al. advised measuring the distances between the floor of the sigmoid groove and a line drawn through the anteromedial surface of the mastoid part of the facial canal on the axial CT images of the temporal bone [4]. Osch et al. built three-dimensional anatomical models from micro-CT images of the cadaveric temporal bones to study the spatial relations between the sigmoid sinus, facial nerve, and external acoustic canal [8]. The easiest method for evaluation of the topography of the sigmoid sinus on magnetic resonance venograms was proposed by Tsutsumi et al.: authors measured the distance between the external acoustic meatus and the sinus and registered its position as superior (11%), intervening (85%), or inferior (4%) [14]. We modified Tsutsumi’s method and measured the distance from the external acoustic canal to the transverse-sigmoid junction specifically and have distributed the obtained data into three groups by the value of the cranial index (Table 2). According to the authors, the inferior position of the sinus coexists with the extreme approximation of the sinus to the external acoustic canal; these data are in line with our results. The correlation of the positional relationships of the sigmoid sinus with the individual features of cranial morphology adds substantial reasoning to the previous research.

The importance of venous anatomy in the selection of the surgical approach is highlighted in the literature. Authors specified that the extreme anterior position of the sinus complicates the operations on the inner ear [4, 13], while the high-posterior position of the sinus requires extended traction of the cerebellum and may cause vascular and neurological complications [9, 10]. Based on anatomic dissection of 94 temporal bones, Singh et al. classified the positioning of the SS into three grades I (favorable), II (intermediate), and III (unfavorable) based on the level of anterior deviation of the sinus [13]. The extreme indentation of the sinus into the temporal bone was registered in 19.15% of the studies specimens, with overall dominance of the second type (41.49%). The authors concluded that the unfavorable (anterior) position of the sigmoid sinus tends to coexist with low pneumatization of the mastoid process. However, the intraoperative prospective study of Galal et al. showed no correlation between the anterior localization of the sigmoid sinus and the degree of the pneumatization of the mastoid process [4]. Obtained in our research correlation between the position of the sigmoid sinus and the diameters of the skull help us to understand the individual variability of the posterior cranial fossa and to forge the morphology-based expectations for the examined structures.

### Limitations of our study

1. The prevalence of females in our brachycephalic MRV group could affect the overage of the results in this group. Additional analysis of the data in the dolichocephalic, mesocephalic, and brachycephalic females only revealed the same patterns of distribution of the variables.
2. The data presented in the present research were taken from multiple populations: we studied the MRV images of Arabic patients and dissected skull bases of Caucasian cadavers. The populational features of cranial morphology were not addressed in this research. The wide range of reported frequency of the low anterior position of the sigmoid sinus (4% in research on Western cohort [14], 19.15% in a study on Indian population [13]) may reflect the dominance of the laterolateral diameter in the Asian population. Additional research on the populational variability of the skull base should be conducted.
3. The larger sample size is advised to be considered in future studies.

### Future perspectives

The morphological data presented in this paper illustrate the possibilities of the clinical application of cranial morphology. Knowledge of the patterns of distribution of the variables by the type of skull allows us to classify the variability of the skull base structures efficiently. In the present study, we focused our attention on the phenotypical variability of the posterior cranial fossa, and positioning of the sigmoid sinus in relation to the internal and external acoustic meatuses; the variability of the orbit, anterior cranial fossae, and the midline structures of the skull base are the potential subjects for the future studies.

## Conclusions

The shape of the skull reflects the morphology of the posterior cranial fossa and influences the topographic characteristics of the sigmoid sinus that must be considered in the selection of surgical approach to the inner ear and pontocerebellar angle. In brachycephalic patients, the translabyrinthine approach to the structures of the inner ear is jeopardized by the high possibility of the low-anterior position of the sigmoid sinus. In dolichocephalic ones, the retrosigmoid approach may require extensive cerebellar retraction and is unfavorable. Thus, the cranial phenotype of the patient should be considered in the planning of the skull base surgery.

## Data Availability

Not applicable.
